# *WNT2* Promotes Cervical Carcinoma Metastasis and Induction of Epithelial-Mesenchymal Transition

**DOI:** 10.1371/journal.pone.0160414

**Published:** 2016-08-11

**Authors:** Yun Zhou, Yongwen Huang, Xinping Cao, Jing Xu, Lan Zhang, Jianhua Wang, Long Huang, Shuting Huang, Linjing Yuan, Weihua Jia, Xingjuan Yu, Rongzhen Luo, Min Zheng

**Affiliations:** 1 Department of Gynecology, Sun Yat-Sen University Cancer Center, 651 Dongfeng Road East, Guangzhou, Guangzhou, 510060, P. R. China; 2 Department of Radiotherapy, Sun Yat-Sen University Cancer Center, 651 Dongfeng Road East, Guangzhou, Guangzhou, 510060, P. R. China; 3 Department of Pathology, Sun Yat-Sen University Cancer Center, 651 Dongfeng Road East, Guangzhou, Guangzhou, 510060, P. R. China; 4 State Key Laboratory of Oncology in South China, 651 Dongfeng Road East, Guangzhou, Guangzhou, 510060, P. R. China; 5 Collaborative Innovation Center for Cancer Medicine, 651 Dongfeng Road East, Guangzhou, Guangzhou, 510060, P. R. China; 6 Cardiovascular Department, Second People's Hospital of Guangdong Province, 1 Shi-liu gang Road East, Guangzhou, 510317, P. R. China; 7 Department of Oncology, the Second Affiliated Hospital, Nanchang University, Nanchang, 330000, P. R. China; China Medical University, TAIWAN

## Abstract

**Background:**

Previously, we found an 11-gene signature could predict pelvic lymph node metastasis (PLNM), and *WNT2* is one of the key genes in the signature. This study explored the expression and underlying mechanism of *WNT2* in PLNM of cervical cancer.

**Methods:**

*WNT2* expression level in cervical cancer was detected using western blotting, quantitative PCR, and immunohistochemistry. Two *WNT2*-specific small interfering RNAs (siRNAs) were used to explore the effects of *WNT2* on invasive and metastatic ability of cancer cells, and to reveal the possible mechanism of *WNT2* affecting epithelial—mesenchymal transition (EMT). The correlation between *WNT2* expression and PLNM was further investigated in clinical cervical specimens.

**Results:**

Both *WNT2* mRNA and protein expression was upregulated in cervical cancer. High *WNT2* expression was significantly associated with tumor size, lymphovascular space involvement, positive parametrium, and most importantly, PLNM. PLNM and *WNT2* expression were independent prognostic factors for overall survival and disease-free survival. *WNT2* knockdown inhibited SiHa cell motility and invasion and reversed EMT by inhibiting the *WNT2*/β-catenin pathway. *WNT2* overexpression in cervical cancer was associated with β-catenin activation and induction of EMT, which further contributed to metastasis in cervical cancer.

**Conclusion:**

*WNT2* might be a novel predictor of PLNM and a promising prognostic indicator in cervical cancer.

## Background

Cervical cancer is the third most commonly diagnosed cancer in women worldwide[1]. Despite the availability of early screening programs, the morbidity of cervical cancer remains high. The morbidity of cervical cancer varies considerably between countries, where more than 77,100 deaths have been recorded in the developing countries[1,2]. Among the clinical prognostic factors for cervical cancer, peripheral lymph node metastasis (PLNM) is one important prognostic factor[3,4]. The rate of PLNM in stage IB–IIA cervical cancer (stage IB, T1BN0M0; stage IIA T2bN0M0; stage IB or IIA is a combination of tumor-nodes-metastasis [TNM] classification that meets the criteria for stage IB or IIA disease) is 22%–27%[4]. The number of PLNM sites is also the most important prognostic factor in cervical cancer. The 5-year overall survival (OS) rate is 93.3%–13.8% in patients with cervical cancer with 1 to ≥4 positive nodes. The OS rate of patients with cervical cancer with 1 or 2 PLNM sites is significantly better than that for patients with >2 PLNM sites[5]. Multivariate analysis has revealed that >2 PLNM sites is an independent prognostic factor for survival[6]. Although the presence of PLNM does not affect staging according to the International Federation of Obstetrics and Gynecology (FIGO) system, it modulates postoperative therapy and affects prognosis. However, computed tomography (CT), magnetic resonance imaging (MRI), and positron emission tomography (PET) preoperative evaluation of nodal status are insufficiently sensitive for detecting small PLNMs (< 1.0 cm in greatest dimension) [7]. Early-stage patients with undetected PLNM, still undergo lymph node dissection, which might lead to infection, increased incidence of complications, and immune system damage[6]. Therefore, searching for promising markers to accurately detect PLNM in patients with early-stage cervical carcinoma is of great clinical importance, as it would aid gynecologic oncologists in directing the choice of treatment, avoiding unnecessary surgical intervention.

In a previous study, we discovered an 11-gene signature that could predict PLNM. In this preliminary study, evaluated expression of *WNT2* was frequently observed in samples with PLNM compared to that without PLNM, and thus stood out as a potential candidate gene in regulating PLNM[8]. The human *WNT* gene family consists of 19 members, which encode evolutionarily conserved glycoproteins with 22 or 24 Cys residues[9]. *WNT* signaling are involved in regulating cell proliferation, differentiation, apoptosis, and migration[10–12]. Recent reports have uncovered the role of *WNT2* in the development and progression of various cancers. For example, *WNT2* is up-regulated in gastric cancers, and could impact tumor formation, invasion and dissemination[13,14]. Tumor fibroblast cells also secrete *WNT2*, which acts as a growth and invasion-promoting factor by activating the canonical *WNT*/β-catenin signaling pathway in esophageal cancer cells[15]. However, the relationship between *WNT2* expression and lymph node metastasis in cervical cancer, and the involved mechanism remains largely unknown.

In this study, we investigated the expression and biological significance of *WNT2* in clinical samples from cervical cancer patients and a cervical cancer cell line. We found that expression of *WNT2* was evaluated in cervical tumor samples, especially in samples with PLNM. The overexpression of *WNT2* was an independent prognostic factor for both OS and DFS. Moreover, downregulation of *WNT2* could inhibit cervical tumor cell motility and invasion, and reversed EMT by inhibiting the *WNT2*/β-catenin pathway.

## Materials and Methods

### Samples and Patients

This study involved 314 patients diagnosed with early-stage cervical cancer (stage Ib–IIa) who underwent radical hysterectomy and lymphadenectomy at the Department of Gynecologic Oncology (Sun Yat-sen University Cancer Center, Guangzhou, China) from January 2002 to December 2006. Thirty fresh cervical cancer tissues, their paired adjacent noncancerous cervical tissues, and normal cervical tissues were collected for qPCR and western blotting. We obtained prior consent from the patients for the research use of the paraffinembedded tissues and fresh tissues. Informed consent was obtained written informed consent from the particients. The Sun Yat-Sen University Cancer Center Institutional Review Board approved this study. The clinicopathological features of the cervical cancer cohort are outlined in Table 1. All patients were followed up regularly, and the last follow-up was carried out in May 2012; the mean observation period was 73 months (1–124 months) and there were 33 cancer-related deaths. All 51 patients in whom lymph node metastasis was detected received adjuvant chemoradiotherapy postoperatively.

**Table 1:** Clinicopathological Characteristics and *WNT2* Expression of the Study Cohort.

Clinical/pathological characteristics	No. of patients (%)
**Age, years**	
<45	185 (59)
≥45	129 (41)
**Squamous cell carcinoma antigen, ng/ml**	
<1.5	120 (38)
≥1.5	58 (18)
**FIGO stage**	
Ib	245 (78)
IIa	69 (22)
**Differentiation grade**	
G1	30 (10)
G2	98 (31)
G3	177 (56)
**Tumor size, cm**	
<4	235 (75)
≥4	78 (25)
**Deep stromal invasion**	
No	148 (47)
Yes	165 (53)
**lymphovascular space invasion**	
No	293 (93)
Yes	21 (7)
**Positive parametrium**	
No	299 (95)
Yes	7 (2)
**Positive surgical margin**	
No	284 (90)
Yes	22 (7)
**Pelvic lymph node metastasis**	
No	239 (76)
Yes	75 (24)
**Recurrence**	
No	267 (85)
Yes	43 (14)
**Expression of WNT2**	
Low or none	154 (49)
High	160 (51)

### Cell Line and Antibodies

The SiHa cervical cancer cell line was obtained from the Shanghai Institutes for Biological Sciences Cell Bank (Shanghai, China), and was tested and authenticated by short tandem repeat genotyping. The cells were cultured in Roswell Park Memorial Institute 1640 medium (GIBCO BRL, Rockville, MD, USA) supplemented with 10% fetal bovine serum (FBS; HyClone Laboratories, Logan, UT, USA). Three human short hairpin RNA (shRNA) sequences for repressing *WNT2* expression were cloned; S1 Table lists their sequences. Retroviral production and infection were performed as described previously[16]. Mouse anti—E-cadherin, anti-vimentin (BD Transduction Laboratories, Lexington, UK), and anti–β-tubulin (Abmart, Shanghai, China), and rabbit anti-*WNT2* (Abcam, Cambridge, UK) and anti–β-catenin (Cell Signaling Technology, Danvers, MA, USA) antibodies were used in this study. Rhodamine-conjugated antibodies (Invitrogen, Carlsbad, CA, USA) and diaminophenylindole (Sigma-Aldrich, St Louis, MO, USA) were used in immunofluorescence analysis.

### RNA Extraction, Reverse Transcription, and qPCR

Total RNA from cultured cells and fresh tissues were extracted using TRIzol (Invitrogen) according to the manufacturer’s instruction. About 2 μg RNA per sample was used for complementary DNA (cDNA) synthesis using an iScript cDNA Synthesis Kit (Bio-Rad Laboratories, Hercules, CA, USA). We used qPCR to quantify the fold change of *WNT2* mRNA levels using a Bio-Rad CFX96 sequence detection system with SsoFast EvaGreen Supermix (Bio-Rad Laboratories). Expression data were normalized to the geometric mean of the housekeeping gene comparative threshold value [2^−ΔΔCt(GAPDH-WNT2)^]. The primer sequences are as follows: *WNT2* forward, 5′-TCCAGACCATTAACCTCAGTGC-3′; *WNT2* reverse, 5′-TGTATTCCATCACCACCAGCC-3′; β-catenin forward, 5′-CTGTCCCAACTGTAAAGAAGGTG-3′; β-catenin reverse, 5′-AGAATGCCAACGCAGATGAG-3′; E-cadherin forward, 5′-GACCGGTGCAATCTTCAAA-3′; E-cadherin reverse, 5′-TTGACGCCGAGAGCTACAC-3′; vimentin forward, 5′-TGGCTCGCATTCATTTTCTG-3′; vimentin reverse, 5′-TGTGGCATCAATGAAGTACCCT-3′; and glyceraldehyde-3-phosphate dehydrogenase (GAPDH) forward, 5′-GACTCATGACCACAGTCCATGC-3′; GAPDH reverse, 5′-AGAGGCAGGGATGATGTTCTG-3′. All genes were tested in triplicate.

### Immunohistochemistry

Immunohistochemical staining was carried out using Histostain-Plus Kits (Invitrogen) according to the manufacturer’s protocol. Two independent pathologists blinded to the clinical parameters conducted the immunoreactivity scoring for *WNT2* expression. The staining results were scored based on the following criteria: (i) percentage of positive tumor cells: 0 (0%), 1 (1%–10%), 2 (11%–50%), 3 (51%–70%), or 4 (71%–100%); (ii) staining intensity: 0 (no signal), 1 (weak), 2 (moderate), or 3 (strong). The immunoreactivity score was calculated by summing the percentage of the positive cell score and the intensity score (score range: 0, 1, 2, 3, 4, 6, 8, 9, 12) [17]. A High expression was defined as a final score ≥ 6; low expression was defined as a final score < 6.

### Tissue Microarray

Hematoxylin and eosin—stained sections from each paraffin-embedded, formalin-fixed block were used to define diagnostic areas; 2–5 (average, 4) random representative 0.6-mm cores were obtained from each case and inserted in a grid pattern into a recipient paraffin block using a tissue arrayer (Beecher Instruments, Silver Spring, MD, USA). Sections (5 μm) were then cut from each tissue microarray and stained with antibodies against *WNT2*, β-catenin, E-cadherin, and vimentin[18].

### Wound Healing, Cell Invasion, and Migration Assays

The SiHa cells were plated to confluence in 6-well plates. Scratches were made in the monolayer with a pipette tip. The progression of cell migration was observed and photographed at 24h after wounding. The cell invasion and migration assays were performed using Transwell chambers (Corning Inc, Corning, NY, USA) with or without Matrigel coating (BD Biosciences, San Diego, CA, USA). The lower chambers were filled with 500 μl DMEM supplemented with 10% FBS. After 24-h incubation, cells invading to the bottom side of the inserts were fixed, stained, photographed, and quantified by counting in five random high-power fields[19].

### Statistical Analysis

Statistical analyses were performed using SPSS v16.0 software (IBM, USA). The relationship between *WNT2* expression and clinicopathological characteristics was assessed using Pearson’s χ^2^ test. Survival curves were plotted using the Kaplan—Meier method and compared using the log-rank test. Multivariate survival analysis was carried out for all parameters that were significant in univariate analysis using the Cox regression model. A two-sided probability value of <0.05 was considered statistically significant.

## Results

### *WNT2* Expression was Elevated in Cervical Cancer

**Fig 1:**
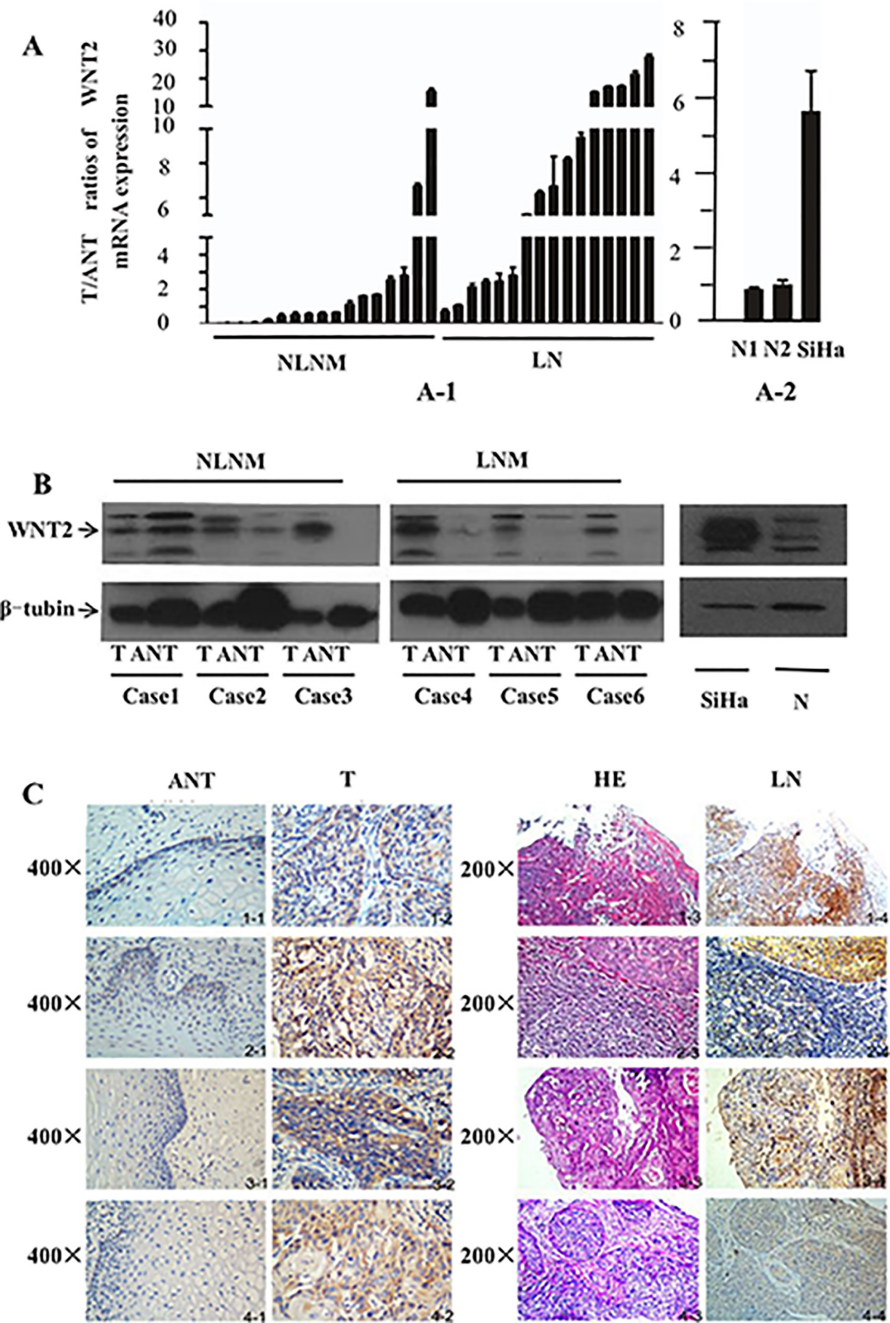
Western blotting, qPCR, and immunohistochemical assay of *WNT2* protein and mRNA expression. Error bars represent standard deviation values calculated from three parallel experiments. (A) (A-1) qPCR assay of *WNT2* expression in tissues from patients with and without lymph node metastasis. LNM, samples with PLNM in postoperative histological review; NLNM, samples without PLNM in postoperative histological review. (A-2) qPCR assay of *WNT2* expression in fresh cervical tissues and the SiHa cell line. T/ANT ratios of *WNT2* mRNA expression were quantified by qPCR in 34 pairs of matched cervical cancer tissues. (B) Western blotting analysis of *WNT2* protein expression in six pairs of matched cervical cancer tissues (T) with and without PLNM and their adjacent noncancerous cervical tissues (ANT). (C) Immunohistochemical assay of *WNT2* protein expression in four pairs of matched cervical cancer tissues with PLNM and matched lymph node (LN) tissues. HE, hematoxylin and eosin.

**Fig 2:**
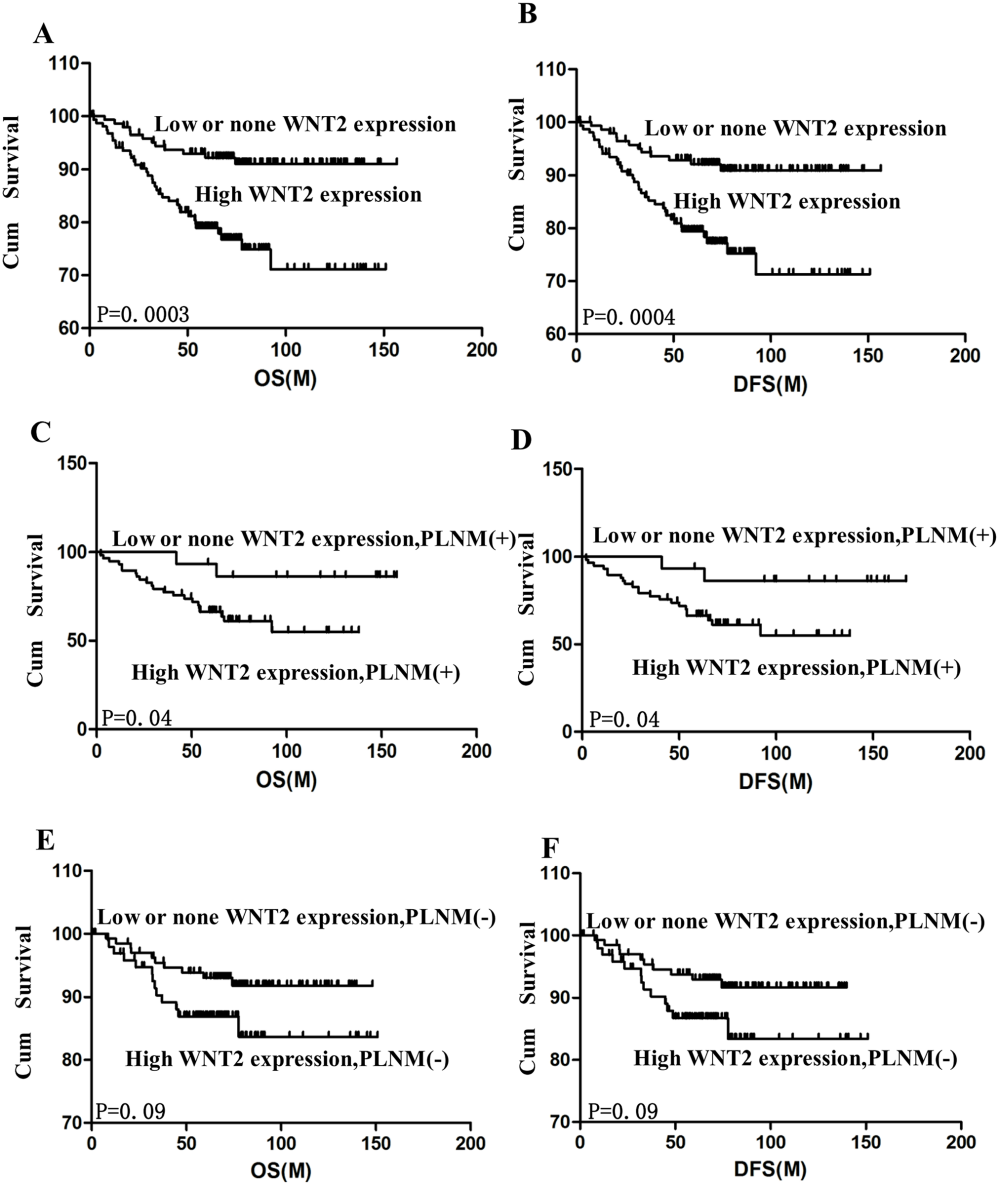
Kaplan-Meier curves obtained from univariate analyses (log-rank) of patients with cervical cancer and low versus high *WNT2* expression (M, Month). (A) OS rate for patients with high versus low *WNT2* expression. (B) DFS rate for patients with high versus low *WNT2* expression. (C and E) OS rate for patients with high versus low *WNT2* expression stratified based on PLNM. (D and F) DFS rate for patients with high versus low *WNT2* expression stratified based on PLNM.

We performed western blotting, qPCR, and immunohistochemistry assay on SiHa cervical cancer cells, fresh cervical tissues, and fresh cervical cancer tissues with paired adjacent noncancerous tissues to investigate the potential role of *WNT2* in the tumorigenesis and lymphatic metastasis of cervical cancer. Both *WNT2* protein expression and mRNA expression were upregulated in the SiHa cells and in cervical cancer specimens compared with that in the adjacent noncancerous tissues (Fig 1A–1C). The T/ANT ratios for *WNT2* mRNA expression levels in the specimens without PLNM (0.00–17.69, Fig 1A) were lower than that in the specimens with PLNM (0.95–28.84, Fig 1A), and *WNT2* mRNA expression was highly expressed in the SiHa cells as compared with the normal cervical tissues (Fig 1A). Western blotting confirmed the protein level of *WNT2* was evaluated in tumors with PLNM (Fig 2B). *WNT2* was upregulated in 51.3% (161/314) of paraffin-embedded cervical cancer tissues. By contrast, *WNT2* was not observable in the marginal areas surrounding the cancerous tissues. Moreover, *WNT2* protein was highly expressed in 91% (20/22) of metastatic lymph node tissues following postoperative pathological confirmation (Fig 1C).

### *WNT2* Expression was Associated with PLNM

Subsequently, we studied the association between *WNT2* expression level and the patient clinicopathological characteristics. There was a significant correlation between *WNT2* expression level and prognostic risk factors such as lymphovascular space involvement (LVSI, *P* = 0.001), positive parametrium (*P* = 0.031), and most importantly, lymph node metastasis (*P* < 0.0001) (Table 2). Moreover, squamous cell carcinoma antigen, an tumor marker for predicting PLNM, was sinificantly correlated with *WNT2* expression level (P = 0.029, Table 2). Univariate and multivariate analyses revealed that *WNT2* expression (P < 0.001) and deep stromal invasion (P = 0.009) were independent factors for PLNM. Table 3 lists the results of Spearman correlation analysis between *WNT2* expression and the clinicopathological factors.

**Table 2:** The Association between *WNT2* Expression Level and Clinicopathological Characteristics of Patients with Early-stage Cervical Cancer.

Clinical/pathological characteristics	No. of patients (%)		Chi-square *P*
	Low or none WNT2 expression	High WNT2 expression	
**Total no. of patients**	153 (49)	161 (51)	
**Age, years**			0.909
<45	91 (29)	94 (30)	
≥45	62 (20)	67 (21)	
**Squamous cell carcinoma antigen, ng/ml**			**0.029**
<1.5	70 (22)	50 (16)	
≥1.5	31 (10)	27 (9)	
**FIGO stage**			0.238
Ib	123 (39)	122 (39)	
IIa	30 (10)	39 (12)	
**Differentiation grade**			0.484
G1	20 (6)	10 (3)	
G2	52 (17)	46 (15)	
G3	81 (26)	96 (31)	
**Tumor size, cm**			**0.0001**
<4	136 (43)	99 (32)	
≥4	16 (5)	62 (20)	**0.0001**
**Deep stromal invasion**			
No	97 (31)	51 (16)	
Yes	56 (18)	109 (35)	
**Lymphovascular space invasion**			**0.012**
No	150 (48)	143 (46)	
Yes	3 (1)	18 (6)	
**Positive parametrium**			**0.031**
No	202 (64)	97 (31)	
Yes	2 (1)	5 (2)	
**Positive surgical margin**			0.247
No	190 (61)	94 (30)	
Yes	14 (4)	8 (3)	
**Pelvic lymph node metastasis**			0.0001
No	138 (44)	101 (32)	
Yes	15 (5)	60 (19)	
**Recurrence**			**0.0001**
No	142 (45)	125 (40)	
Yes	11 (4)	32 (10)	
**Vital status at follow-up**			0.001
Alive	134 (43)	122 (39)	
Death from CC	12 (4)	35 (11)	

Bold values indicate Chi-square P < 0.05.

**Table 3:** Spearman Correlation Analysis between *WNT2* Expression Level and Clinicopathologic Factors.

Variables	WNT2 expression level	
	Spearman Correlation	*P*-value
**Tumor Size**	0.323	< 0.001
**Squamous cell carcinoma antigen**	0.148	0.049
**Lymphovascular space involvement**	0.316	< 0.001
**Positive parametrium**	0.184	0.001
**Pelvic lymph node metastasis**	0.322	< 0.001
**Recurrence**	0.191	0.001

### *WNT2* Expression Predicted Poor Survival

Kaplan-Meier analysis was used to investigate whether *WNT2* expression was of prognostic significance. In our study cohort, high *WNT2* expression was significantly associated with both shorter OS (*P* < 0.001, log-rank test) and disease-free survival (DFS; *P* < 0.001, log-rank test; Fig 2A and 2B). The 10-year OS and DFS rates were 73% and 72%, respectively, in patients with high *WNT2* expression as compared to that in patients with low *WNT2* expression, in whom both the OS and DFS rate was 91%. Furthermore, when stratified based on PLNM, both the OS and DFS rates of patients with high *WNT2* expression were significantly lower than that of patients with low or no *WNT2* expression (both *P* < 0.001, log-rank test; Fig 2C–2F). The Cox regression model revealed that PLNM (hazard ratio [HR]: 2.497, 95% confidence interval [CI] 0.915–8.135, *P* = 0.004) and *WNT2* expression level (HR: 2.404, 95% CI 0.877–6.617, *P* = 0.018) were independent prognostic factors for poor OS. Consistent with that, PLNM (HR: 2.485, 95% CI 0.910–5.636, *P* = 0.004) and *WNT2* expression level (HR: 2.410, 95% CI 0.880–6.617, *P* = 0.018) were independent prognostic factors for poor DFS.

### *WNT2* Knockdown Inhibited SiHa Cell Motility and Invasion

**Fig 3:**
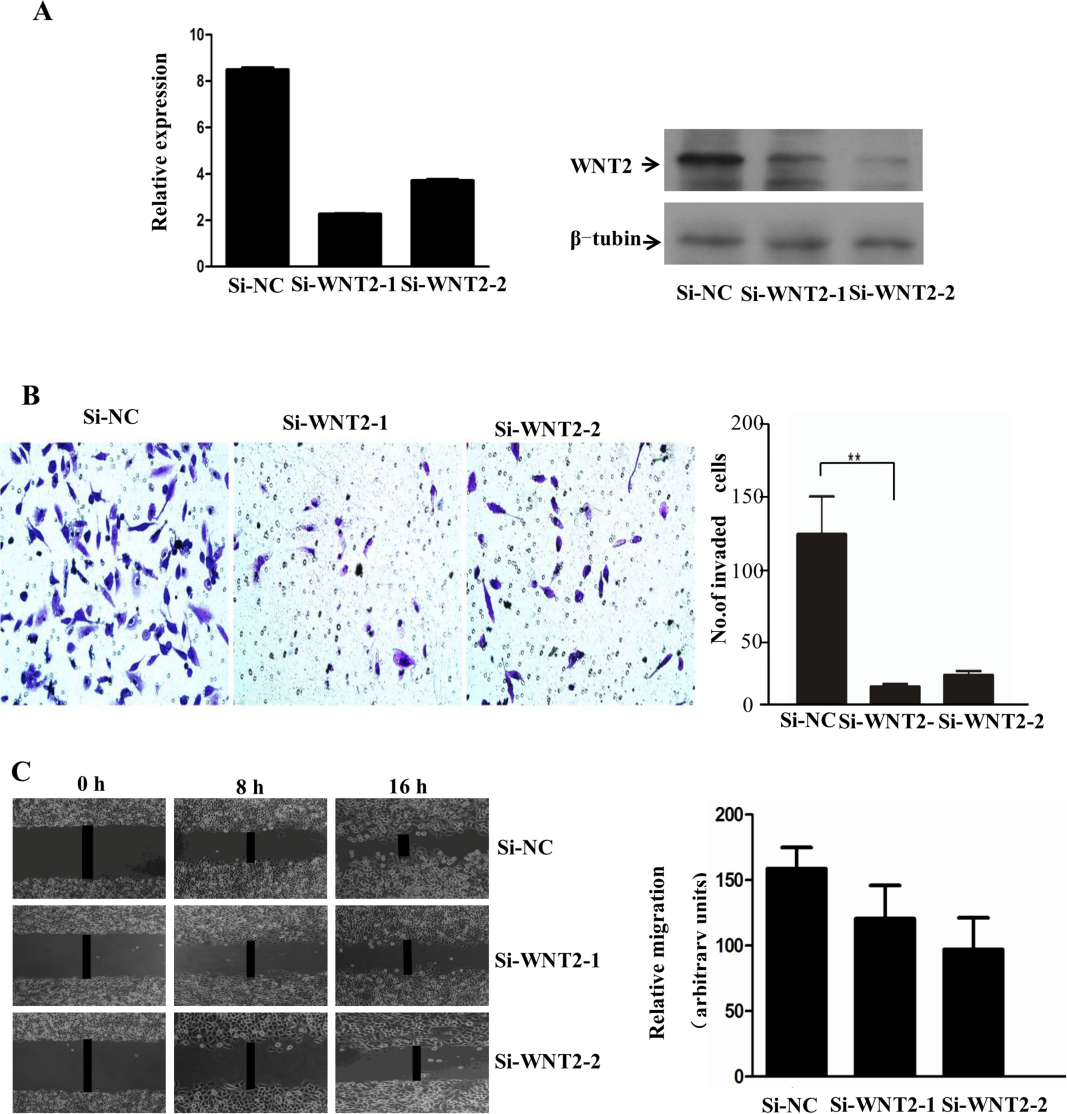
Downregulating *WNT2* inhibited cell motility and invasion. (A)Knockdown of *WNT2* inhibited the expression of *WNT2* both in mRNA and protein expression levels. (B) Invasion assay analysis of FBS-induced invasive properties (×200, **P < 0.001). Scramble: cells transfected with scramble shRNA (siRNA); siRNA/siRNA1 and 2: cells transfected with two human shRNA sequences to repress *WNT2*. (C) Cell mobility was measured by examining the rate of wound closure at 0, 8, and 16 h (×200), *WNT2* knockdown decreases in vitro migration in a scratch assay. shRNA (siRNA);siRNA1 and 2 cells were seeded into six-well plates overnight and scratched the next day. Images were collected at the time points listed. Relative migration was quantified by measuring the distance between scratch edges for 10 different points and compared with time 0.

We used two *WNT2*-specific siRNAs to explore the functional significance and possible mechanism of *WNT2* in the invasive capability of SiHa cells, knockdown of *WNT2* inhibited the expression of *WNT2* both in mRNA and protein expression levels (Fig 3A). Boyden chamber invasion assay revealed that the motility and invasiveness of SiHa cells were dramatically hampered by the ablation of *WNT2*; the average number of Siha cell transfected with short hairpin RNA-1 and RNA-2 were respectively 17 and 26, which was more than Siha cell transfected without short hairpin RNA. Cell mobility was measured by calculating the rate of wound closure at 0, 8, and 16 h in ×200 magnification. After 16h, the distance between shRNA-transfected SiHa cells on both sides of the wound was shorter than that between shRNA-untransfected SiHa cells (Fig 3B and 3C). As expected, silencing *WNT2* led to decreased cell motility and invasiveness.

### *WNT2* Downregulation Reversed EMT and Suppressed the *WNT2/β-Catenin* Pathway

**Fig 4:**
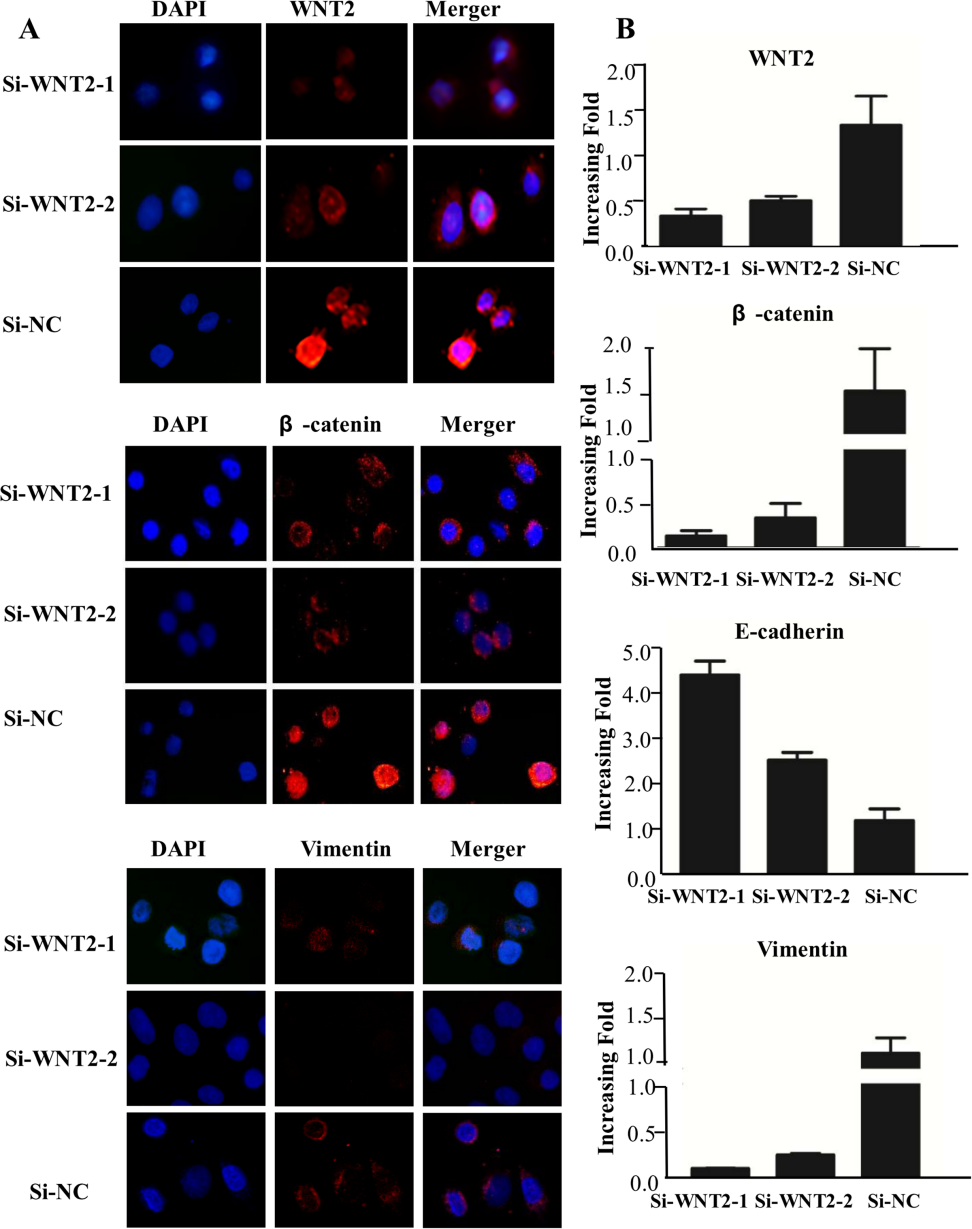
Downregulating *WNT2* reversed EMT and inhibited the *WNT2*/β-catenin pathway. (A) Immunofluorescence analysis of *WNT2*/β-catenin pathway proteins and EMT markers (×100). (B) qPCR analysis of the expression of EMT markers and *WNT2*. Glyceraldehyde 3-phosphate dehydrogenase was the loading control.

Although, there were no EMT-associated morphological changes detected in *WNT2* knockdown SiHa cells, the inhibition of endogenous *WNT2* suppressed the expression of epithelial markers such as E-cadherin, but partially rescued the expression of mesenchymal markers such as vimentin and fibronectin. Both qPCR and immunofluorescence showed that E-cadherin expression was decreased in *WNT2*-suppressed SiHa cells; however, vimentin expression was increased (Fig 4A and 4B). SiRNA silencing of *WNT2* expression inhibited *WNT2*/β-catenin pathway activity. The knockdown of endogenous *WNT2* reduced EMT in the SiHa cells, which was observed as decreased E-cadherin expression and increased vimentin expression, and prevention of β-catenin/E-cadherin complex formation.

### Clinical Association of *WNT2* Expression with PLNM in Human Cervical Cancer

**Fig 5:**
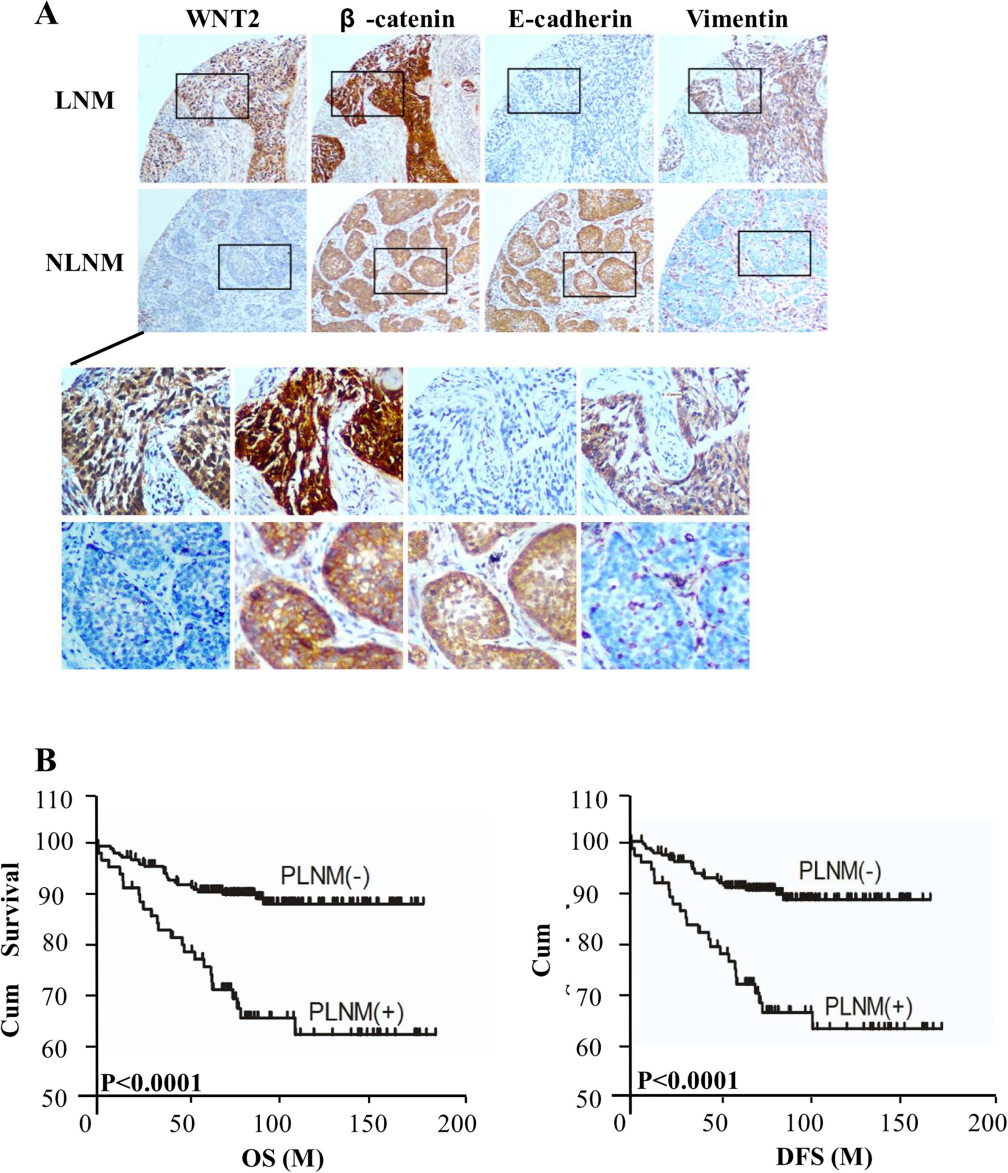
Clinical association of *WNT2* expression with PLNM in cervical cancer. (A) PLNM was associated with *WNT2* expression and β-catenin, E-cadherin, and vimentin localization in cervical cancer specimens. (B) Kaplan-Meier metastasis-free survival curve comparing groups with and without PLNM in the primary tumors of 314 patients with cervical cancer; the *P*-value is based on log-rank testing. (M, Month) (C) Percentage of specimens with or without PLNM in relation to *WNT2*, β-catenin, E-cadherin, and vimentin expression levels. *P < 0.05.

To further investigate the clinical relevance of the above findings in human cervical cancer, we examined *WNT2* expression and EMT features in 314 human cervical cancer tissue specimens. Patients were first divided into those who developed PLNM and those who were PLNM-free within the follow-up period. The expression level of *WNT2* was significantly elevated in the primary tumors of 60 patients with PLNM as compared with that in the primary tumors from 101 patients without PLNM (Fig 5A). Kaplan-Meier analysis showed that PLNM was significantly associated with both shorter OS (*P* < 0.0001, log-rank test) and DFS (*P* < 0.0001, log-rank test; Fig 5B). Moreover, cervical cancer samples with PLNM had higher β-catenin activation (63/75; 84.2%) than PLNM-free samples (122/239; 51%; *P* < 0.05). Importantly, there was strong E-cadherin expression in 132 of 239 specimens without PLNM (55.2%); in contrast, high E-cadherin expression was observed in only 18 of 75 samples with PLNM (23.4%; Fig 5C). Concurrently, there was an inverse expression pattern of vimentin in the same patient cohort, suggesting PLNM is significantly correlated (*P* < 0.05) with *WNT2* expression (Fig 5C). Taken together, *WNT2* overexpression in cervical cancer is associated with β-catenin activation and induction of EMT, which further contributes to the metastasis of cervical cancer.

## Discussion

In this study, we demonstrate that both mRNA and protein level of *WNT2* expression is upregulated in cervical cancer and in comparison with that in the paired adjacent noncancerous cervical tissues for the first time. There was a significant associstion between high *WNT2* expression and tumor size, LVSI, positive parametrium. Most importantly, PLNM and *WNT2* expression level were independent prognostic factors for poor OS and DFS rates in patients with early-stage cervical cancer. Moreover, knockdown *WNT2* led to decreased cell motility and invasiveness reversed EMT by suppressing the *WNT2*/β-catenin pathway. In view of these findings, we believe that *WNT2* is a potential novel predictor of PLNM and a promising therapeutic target in cervical cancer.

So far, the standard treatment for early-stage cervical cancer is radical hysterectomy plus lymphadenectomy or chemoradiation, which have similar survival rates[20–23]. Chemoradiotherapy is required for patients with lymph node metastasis, which renders the initial surgical procedure unnecessary in retrospect[24]. However, as it is not possible to clinically detect pelvic and para-aortic lymph node metastasis accurately and efficiently, gynecologic oncologists cannot direct the choice of treatment, and avoid unnecessary surgical intervention and reduce morbidity[24]. The use of MRI and CT in recent years for determining lymph node status may have been inaccurate; meta-analysis found that the pooled positive likelihood ratio for PET, MRI, and CT was 15.3, 6.4, and 4.3, respectively[25]. The sentinel concept is another promising technique, but a multi-center study reported its low sensitivity[26].

Our study demonstrated that high *WNT2* expression levels were significantly correlated with poor prognostic risk factors and recurrence, especially PLNM. This is consistent with previous clinical observations where *WNT2* expression was associated with increased risk of metastasis and worse prognosis[13,14].

The accuracy of predicting PLNM by observing *WNT2* expression (high versus low) in our cohort was 36% (60/166). However, the recurrence rate was 10.8% in patients with high *WNT2* expression but in whom lymph node metastasis was not observed, whereas recurrence was investigated in only 2.6% of patients (P < 0.01) in the low *WNT2* expression group. As the reported pelvic lymph node micrometastasis rate is 15% in patients with early-stage cervical cancer[27,28], it is highly possibly that the actual accuracy of predicting PLNM could be 51% (36% + 15%) by detecting *WNT2* expression. However, in the present study, only 75 of 314 (23.8%) patients were diagnosed with lymph node metastasis. To address this discrepancy, a further study extended to a larger cohort of patients with lymph node metastasis is currently undertaken, and with micrometastasis taken into account. In our cohort, it is more meaningful that the experiment found high *WNT2* expression in 20 cases of lymph node metastasis, where the positive expression rate was 91% (20/22).

EMT is involved in a number of developmental events and contributes to tumor progression, invasion, metastasis, and dissemination[29]. The mechanism of cancer metastasis has been intensively studied recently and it may provide vital therapeutic targets for preventing metastasis. The major signaling pathways involved in EMT include the transforming growth factor-beta (TGF-β), *WNT*, Notch, and Hedgehog pathways[30]. EMT has been implicated in primary tumor metastasis in cervical cancer and the molecular mechanisms have been described[31]. Cervical cancer cells undergo EMT, and invasion and metastasis are promoted in response to stimulation from overexpressed EMT activators. The mechanism of EMT are divided in four major groups: (i) receptor-mediated signaling by TGF-β1, epidermal growth factor (EGF), and α5β1 integrin, and Jagged1-specific Notch signaling; (ii) an ion transport system containing KCl cotransporter-3; (iii) cytoskeletal modulators comprising gelsolin, and Rho C signaling; (iv) EMT-related transcription factors, including Six1 homeoprotein, Snail, and Twist[31].

Hence, we showed that suppressing *WNT2* decreases cytoplasmic and nuclear accumulation of β-catenin in human cervical cells, downregulating *WNT2* in cervical cancer cells removes the β-catenin/E-cadherin association, and that decreasing β-catenin leads to the disappearance of E-cadherin from the cell surface. We observed this based on the ability of *WNT2* to specifically induce β-catenin cytoplasmic and nuclear localization by regulating β-catenin/E-cadherin interaction. Moreover, downregulating *WNT2* remarkably inhibited cell motility and invasion and reversed EMT by inhibiting the *WNT2*/β-catenin pathway. Consistent with our findings, previously reported findings suggest that *WNT2* expression in human colorectal carcinoma and esophageal cancer may also have a biological function[13–15,32–33].

Finally, we present a clinical correlation, where high *WNT2* expression in human cervical cancer tissue specimens strongly correlates with cytoplasmic and nuclear accumulation of β-catenin and increased metastasis potential. *WNT2* might be an identifying biomarker of PLNM for early cervical cancer, and a new potential therapeutic target.

## Supporting Information

S1 Table The sequences of the shRNA used in the study. (DOC)

## Abbreviations

PLNM: pelvic lymph node metastasis
EMT: epithelial–mesenchymal transition
TNM: tumor-nodes-metastasis
OS: overall surviva
FIGO: International Federation of Obstetrics and Gynecology
CT: computed tomography
MRI: magnetic resonance imaging
PET: positron emission tomography
cDNA: complementary DNA
EGF: epidermal growth factor
