# Supplementary material for: WNT2 Promotes Cervical Carcinoma Metastasis and Induction of Epithelial-Mesenchymal Transition
Source: PLoS One. 2016 Aug 11;11(8):e0160414. doi: 10.1371/journal.pone.0160414 (PMC4981407; doi:10.1371/journal.pone.0160414)
Supplement: S1 Table — (DOC) [file pone.0160414.s001.doc]

**Table S1. The Sequences of the** shRNA Used in the Study

| **Name** | **Sequence（5’→3’）** |  |
| --- | --- | --- |
| si-NC | UUCUCCGAACGUGUCACGUdTdT |  |
| si-WNT2-1 001 | CCAACAGAUUAUCACAAAU dTdT |  |
| si-WNT2-2 002 | GGCUGGUGGUGAUGGAAUA dTdT |  |
| si-WNT2-3 003 | GCGGCAGGUGGAGCCUUCACU dTdT |  |
